# In-hospital mortality among patients injured in motor vehicle crashes in a Saudi Arabian hospital relative to large U.S. trauma centers

**DOI:** 10.1186/s40621-014-0021-4

**Published:** 2014-08-27

**Authors:** Suliman Alghnam, Mari Palta, Azita Hamedani, Patrick L Remington, Mohamed Alkelya, Khalid Albedah, Maureen S Durkin

**Affiliations:** 1 Postdoctoral Researcher, Johns Hopkins Bloomberg School of Public Health, Baltimore, MD USA; 2 King Abdullah International Medical Research Center, King Saud Bin Abdulaziz University for Health Sciences, KAIMRC, KSAU-HS, Riyadh, Saudi Arabia; 3 Population Health Sciences, University of Wisconsin-Madison, Madison, WI USA; 4 Emergency Medicine, University of Wisconsin-Madison, Madison, WI USA; 5 Research Scientist, King Abdullah International Medical Research Center, King Saud Bin Abdulaziz University for Health Sciences, KAIMRC, KSAU-HS, Riyadh, Saudi Arabia; 6 Consultant Surgeon, Department of Surgery, King Abdulaziz Medical City, Riyadh, Saudi Arabia

**Keywords:** In-hospital, Motor vehicle, Mortality, Saudi Arabia, Comparison

## Abstract

**Background:**

Traffic-related fatalities are a leading cause of premature death worldwide. According to the 2012 report *the Global Burden of Disease 2010*, traffic injuries ranked 8th as a cause of death in 2010, compared to 10th in 1990. Saudi Arabia is estimated to have an overall traffic fatality rate more than double that of the U.S., but it is unknown whether mortality differences also exist for injured patients seeking medical care. We aim to compare in-hospital mortality between Saudi Arabia and the United States, adjusting for severity and demographic variables.

**Methods:**

The analysis included 485,611 patients from the U.S. National Trauma Data Bank (NTDB) and 5,290 patients from a trauma registry at King Abdulaziz Medical City (KAMC) in Riyadh, Saudi Arabia. For comparability, we restricted our sample to NTDB data from level-I public trauma centers (≥400 beds) in the U.S. Multiple logistic regression analyses were performed to evaluate the effect of setting (KAMC vs. NTDB) on in-hospital mortality after adjusting for age, sex, Triage-Revised Scale (T-RTS), Injury Severity Score (ISS), mechanism of injury, hypotension, surgery and head injuries. Interactions between setting and ISS, and predictors were also evaluated.

**Results:**

Injured patients in the Saudi registry were more likely to be males, and younger than those from the NTDB. Patients at the Saudi hospital were at higher risk of in-hospital death than their U.S. counterparts. In the highest severity group (ISSs, 25–75), the odds ratio of in-hospital death in KAMC versus NTDB was 5.0 (95% CI 4.3-5.8). There were no differences in mortality between KAMC and NTDB among patients from lower ISS groups (ISSs, 1–8, 9–15, and 16–24).

**Conclusions:**

Patients who are severely injured following traffic crash injuries in Saudi Arabia are significantly more likely to die in the hospital than comparable patients admitted to large U.S. trauma centers. Further research is needed to identify reasons for this disparity and strategies for improving the care of patients severely injured in traffic crashes in Saudi Arabia.

**Electronic supplementary material:**

The online version of this article (doi:10.1186/s40621-014-0021-4) contains supplementary material, which is available to authorized users.

## Background

Traffic-related fatalities are a leading cause of premature death worldwide. An estimated 1.2 million individuals are killed in road crashes globally each year. About 90% of these deaths occur in developing countries, although fewer than half of the world’s vehicles are registered in these countries (Peden et al. [2004]). Like many developing countries, Saudi Arabia has struggled with an excess of traffic deaths for decades (Al Ghamdi [2002a]; Al Ghamdi [2002b]; Al-Naami et al. [2010]; Alghnam et al. [2014]; Ansari et al. [2000]; Barrimah et al. [2012]; Nofal and Saeed [1997]). Despite the fact that official statistics tend to underestimate the burden of traffic fatality in Saudi Arabia, (Barrimah et al. [2012]) they report a traffic fatality rate more than double that of the U.S. (Chan [2013]). Several factors likely contribute to the excess traffic fatality in Saudi Arabia, including a high incidence of traffic crash injuries, higher severity, and deficits in healthcare quality, particularly trauma care. Limited data on the burden of injuries in Saudi Arabia is an additional factor that may contribute to the persistence of Saudi Arabia ’s excess traffic fatality. (Al-Naami et al. [2010]; Mock et al. [2004]; Wisborg et al. [2011]) Lack of information can lead to lack of recognition of traffic fatality as a public health concern, and place traffic crashes as a low priority in governmental agendas.

The United States has been successful in reducing traffic fatalities by both improving trauma care and enacting injury prevention strategies (Guan [2006]; MacKenzie et al. [2006]; Nathens et al. [2000a]; Nathens et al. [2000b]). Comparing in-hospital mortality after injury between these two countries may help quantify the extent to which the excess traffic mortality in Saudi Arabia is due to differences in hospital care, and point to opportunities for quality improvement (Boulanger et al. [1993]; Gómez de Segura Nieva et al. [2009]; Hildebrand et al. [2005]; Jenkinson [1999]; Roudsari et al. [2007]; Tan et al. [2012]). Previous studies of cardiac and high-risk surgery outcomes have suggested that providing healthcare settings with information on their risk-adjusted outcomes is associated with subsequent reductions in mortality and morbidity (Hannan [1994]; Khuri [2002]; O’Connor and The Northern New England Cardiovascular Disease Study Group [1996]).

Because little is known about differences in in-hospital mortality due to trauma between Saudi Arabia and other countries, this study aims to compare in-hospital mortality between Saudi Arabia and the U.S. adjusting for injury severity and demographic variables. Our retrospective analysis of datasets assembled from a large Saudi and multiple U.S. trauma centers employs a direct approach to compare in-hospital mortality across settings.

## Methods

### Saudi hospital characteristics

King Abdulaziz Medical City (KAMC) is a hospital located in Riyadh, the capital of Saudi Arabia. Thirty percent of traffic crashes in Saudi Arabia occur in or near Riyadh. (Riyadh [2009]) KAMC is one of the largest hospitals in Riyadh with over 700 beds. KAMC also has a 132-bed Emergency Department (ED). This hospital serves primarily eastern metropolitan Riyadh and its surrounding areas within the province of Riyadh.

KAMC provides free healthcare, including all medical procedures and medications, for National Guard employees and their families. Patients not affiliated with the National Guard System receive free healthcare if they seek medical attention through the ED. As a result, the ED receives a large number of patient visits each year, exceeding 200,000 in 2010 ([2011]). About 35% of all ED visits lead to hospital admission. KAMC is equipped to treat complex trauma cases 24 hours a day, providing care from specialized teams including emergency physicians, and general, and orthopedic surgeons. Based on published guidelines (Nathens et al. [2004]; Tintinalli et al. [2010]), resources at KAMC resemble those at level I trauma centers in the United States. KAMC has accreditation under the Joint Commission International standards with excellent performance since December 2006. In addition, KAMC has been designated by the American College of Surgeons as a provider of training in *Advanced Trauma Life Support* in Saudi Arabia since 1990 (Alkhatib [2009]).

### Datasets

This is a retrospective analysis using two existing datasets: the KAMC Saudi Trauma Registry and the U.S. National Trauma Data Bank (NTDB).

#### Saudi trauma registry

The Saudi Trauma Registry is a prospectively recorded database initiated in 2001. An injured patient must meet at least one of the following criteria to be included: (1) admission to the hospital ward or intensive care unit from the ED; (2) transfer to urgent surgery from the ED; (3) indirect admission (patient discharged from ED and asked to return later); or (4) death after arrival to the ED.

A structured data checklist is used to gather information on patient demographic, physiologic (i.e. Triage-Revised Trauma Scale (Baker and Li [2012]), anatomic (i.e. Injury Severity Score (Schluter [2011a]), and outcome variables. A nurse fills in the checklist and a trained research coordinator ensures that it is complete, tracks missing data, and enters the information into the registry using *Microsoft® Access* software. Data on post-discharge visits and information about co-morbidities are not included. Some of the variables available in this dataset are the following: Demographics (age, sex), mechanism of injury (motor vehicle crash, fall, motorcycle, violence), severity measures, hospital length of stay, and disposition.

#### U.S. National Trauma Data Bank (NTDB)

The NTDB, managed by the American College of Surgeons, is the largest trauma dataset ever assembled in the U.S (Haider et al. [2012b]; Haider et al. [2012a]; Haider et al. [2009]). Information in this registry is voluntarily reported by more than 700 trauma centers and hospitals in the United States and its territories. It includes detailed information on type, location, and severity of injuries as well as patient demographics. The NTDB also contains information on procedures performed as well as patient discharge disposition. Observations mostly come from level I or II trauma centers, where more resources are available to meet urgent needs, such as specialized surgery. Therefore, trauma care is expected to be more advanced and well equipped than in other healthcare settings (i.e. a level IV trauma center) (Guan [2006]; Nathens et al. [2004]).

The inclusion criteria (Neal [2013]) for the NTDB are: *International Classification of Diseases, Ninth Revision, Clinical Modification (ICD-9-CM)* discharge diagnosis 800.00–959.9 and either (1) admission; (2) transfer via EMS transport (including air ambulance) from one hospital to another hospital, or (3) death after receiving any evaluation or treatment; or being dead on arrival (Neal [2013]). Patients with the following ICD-9-CM discharge diagnoses are excluded from this dataset: 905–909 (late effects of injury), 910–924 (blisters, contusions, abrasion, and insect bites), and 930–939 (foreign bodies).

### Patient population and selection

This study focuses specifically on patients injured in traffic related crashes. A crash is defined as any traffic-related collision involving a motorized or non-motorized vehicle including: single vehicle (car/bicycle/motorcycle), two vehicles or more, or a pedestrian struck by a vehicle. The KAMC’s registry did not have a separate category for bicyclists and included them with pedestrians because bicycling is rare in Saudi Arabia. However, since the NTDB uses ICD-9 to classify causes of injuries, bicycling can be retained as separate injury mechanism. We chose to keep bicycling as a separate mechanism because grouping them with pedestrians as was done in Saudi Arabia would have reduced comparability of the pedestrian category. Other approaches (e.g. exclusion, inclusion with pedestrians) did not change findings.

Patients in KAMC are included in this analysis if they were seen in the ED between the years 2001 and 2010. The U.S. sample was obtained from the NTDB for the years 2002–2010. In the U.S. dataset, an ICD-9 E code is used to classify injury. We included patients who had ICD-9 external causes of injury in the range of E810-E819, which indicates a traffic-related cause. This approach was based on the recommended framework for injury and mortality data of the Center of Disease and Control (Haider et al. [2009]). No information about patient re-admission was available in either of the datasets.

Because the Saudi registry comes from a large public hospital, we limited the comparison group (U.S.) to 162 public, level I hospitals (≥400 beds). Trauma-center levels in the U.S. were based on designation by states or verification by the American College of Surgeons (MacKenzie et al. [2006]).

### Outcome of interest

The primary outcome is death in the emergency department or during the hospital stay. ED deaths are those who arrived alive and had baseline assessment data (i.e. SBP) then died while death on arrival (DOA) were patients who had no baseline vitals and as a result were excluded from the analysis.

### Statistical analysis

STATA version 12 for Mac (STATA Corp., College Station, TX) was used for all statistical analyses. To examine differences in health outcome across the two settings, the datasets were combined into a single analysis file. Patients were compared on the following variables: age, sex, mechanism of injury, Triage-Revised Trauma Scale (T-RTS), Injury Severity Score (ISS), presence of head injuries, surgery, and hypotension at admission (systolic blood pressure <90 mm Hg.). Student’s t test was used to compare continuous variables and Chi-square tests to compare categorical variables and proportions between Saudi Arabia and the U.S. Because there are documented differences within the U.S. in trauma outcomes (Shafi et al. [2010]; Shafi et al. [2009]), we divided in-hospital mortality into deciles and plotted how KAMC ranks relative to other hospitals in the overall distribution.

Unadjusted and adjusted mortality were compared between settings using logistic regression with an indicator variable for setting. The following variables were included in the multivariable analysis: age, sex, ISS, T-RTS, mechanism of injury (motor vehicle occupant versus pedestrian, or motorcyclist), an indicator for transfer to surgery from the ED, an indicator for head injury, and an indicator for being hypotensive (Glance et al. [2012]; Haider et al. [2012b]; Kimura et al. [2012]; Schluter [2011a]). Based on prior literature ISS was entered into the model as a categorical variable (1–8, 9–15, 16–24 and 25–75) (Haider et al. [2012b]; Schluter [2011b]). To identify potential subgroups with higher or lower difference between settings, we tested for interaction effects. The results are presented as odds ratios (OR) with ninety-five percent confidence intervals. A sensitivity analysis was performed excluding individuals who died in the ED prior to hospital admission. For variables with interaction effects, adjusted odds ratios with 95% confidence intervals are shown for relevant subgroups.

### Missing observations

The Saudi dataset contains very few missing observations (<1%). In the U.S. NTDB, 85% of all patients had complete information and the majority of those with missing information (~12%) were missing either one or two variables. The use of multiple imputation (Galvagno et al. [2012]; Glance et al. [2009]; Haider et al. [2012a]; Moore et al. [2012]; Oyetunji et al. [2011]) did not change our findings, therefore, we chose to present the complete case analysis.

#### Ethical review

This study was reviewed and approved by both the Institutional Review Board at King Abdulaziz Medical City and the University of Wisconsin-Madison Health Sciences Institutional Review Board.

## Results

### Patient characteristics

**Figure 1:**
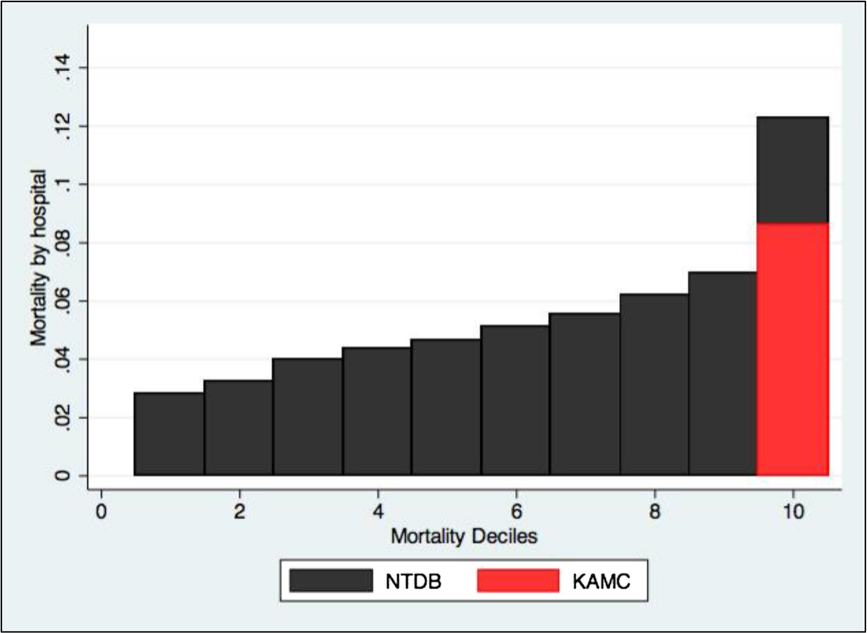
**In-hospital mortality by deciles in hospitals from the National Trauma Data Bank (NTDB) and King Abdulaziz Medical City (KAMC).**

**Figure 2:**
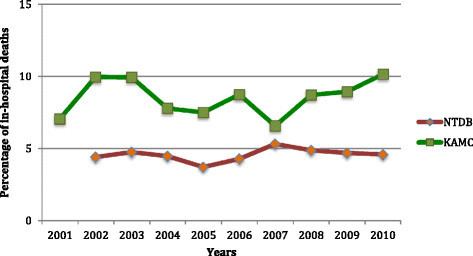
**In-hospital mortality in King Abdulaziz Medical City (KAMC) and level I public trauma centers from the National Trauma Data Bank (NTDB) over the study period.**

A total of 5,290 patients from KAMC and 485,611 from the NTDB were included in the analysis. There were several differences in patient characteristics between settings (Table 1). The overall population of KAMC was significantly younger than that of the NTDB (60.1% 25 years or younger vs. 34.2%, p < 0.001). In addition, the proportion of males in KAMC was higher than in the U.S. (85.5% vs. 63.4%, p < 0.001). The overall mean of the ISS indicated worse status of patients admitted to KAMC than patients from the NTDB although the difference was small. There were 21 KAMC patients with an ISS = 75 (unsurvivable score), who all died in the hospital. On the other hand, only 44% of those with a similar score died among NTDB patients, and our findings were not very different when patients with ISS = 75 were excluded. In addition, the proportion of hypotensives was significantly higher in KAMC than the NTDB. Four hundred forty-three (8.5%) patients in KAMC and 20,928 (4.3%) in the NTDB died at the hospital (Table 1). Mortalities by hospital in the NTDB ranged from 0% to 12.2% with only 3 hospitals having mortality higher than KAMC (Figure 1). The difference in mortality between KAMC and the NTDB was present across study years, as illustrated in Figure 2.

**Table 1:** **Characteristics of traffic-related patients at King Abdulaziz Medical City (KAMC) and National Trauma Data Bank (NTDB)**

Variable	KAMC registryN = 5,290	NTDB N = 485,611
**Age category %**		
**0-14**	20.4	6.8
**15-25**	39.8	28.0
**26-45**	27.9	32.5
**46-64**	7.9	22.1
**≥65**	3.9	10.5
***Missing %***	*0*	*1.3*
***Male %***	85.5	63.4
***Missing %***	*0*	*0.6*
**Role %**		
***Occupant***	73.3	87.9
***Motorcycle***	4.3	5.8
***Pedestrians***	22.4	5.0
***Bicyclists***	A	1.1
**Mean T-RTS [Median, IQR]**	11.0 [12,1.6]	10.9 [11,1.8]
***Missing %***	*0*	*7.7*
**Mean ISS [Median, IQR]**	12.9 [9,11.3]	12.1 [9,10.8]
***Missing %***	*0.1*	*7.5*
***ISS Categories***		
(1–8) N (%)	2,040 (38.5)	209,574 (43.1)
(9–15) N (%)	1,693 (32.0)	132,347 (27.2)
(16–24) N (%)	687 (13.0)	85,222 (17.5)
(25–75) N (%)	870 (16.4)	58,468 (12.0)
**SBP < 90 %**	4.4	3.8
***Missing %***	*0.7*	*2.8*
**Surgery %**	20.8	12.4
***Missing %***	*0*	*1.0*
**Head injury %**	42.5	17.1
***Missing %***	*0*	*0*
**Died in the hospital %**	8.3	4.3
***Missing %***	*0*	*1.9*

All differences significant at p < 0.001,except for SBP at p < 0.01.

^A^Bicyclists are added to pedestrians in King Abdulaziz Medical City (KAMC) registry.

*Chi-2 test; ^Student t-test.

ISS Injury Severity Score; T-RTS Triage-Revised Trauma Scale.

SBP Systolic Blood Pressure.

### Regression results

Both unadjusted and adjusted analyses estimated in-hospital mortality to be higher in KAMC than in the NTDB (Tables 2 and 3). Interaction effects indicated that as ISS increased, so did the odds ratio of in-hospital death in KAMC versus the U.S (p < 0.001). Odds ratios were also significantly higher in older age groups (p < 0.001) and for those with versus without head injuries (p < 0.001). (Table 3).

**Table 2:** **Estimated Odds Ratios (OR) and 95% confidence intervals (CI) from a multivariable logistic regression of predictors of in-hospital mortality among trauma patients from King Abdulaziz Medical City (KAMC) and the National Trauma Data Bank (NTDB)**

Variable	Complete case model (N = 490,901)	Excluding deaths in the ED (N = 446,687)
	OR (95% CI)	OR (95% CI)
**Age category**		
0-14	**Reference**	**Reference**
15-25	1.1 (1.0-1.2)	1.1 (1.0-1.2)
26-45	1.2 (1.1-1.3)	1.2 (1.1-1.3)
46-64	2.2 (2.0-2.4)	2.2 (2.0-2.4)
≥65	9.2 (8.4-10.0)	9.0 (8.1-9.9)
***Male***	1.1 (1.0-1.1)	1.1 (1.0-1.1)
**Role**		
*Occupant*	**Reference**	**Reference**
*Motorcycle*	0.9 (0.8-1.0)	0.9 (0.9-1.0)
*Pedestrians*	1.4 (1.3-1.5)	1.5 (1.1-1.5)
*Bicyclists*	1.0 (0.8-1.2)	1.2 (1.0-1.4)
**T-RTS**	0.6 (0.6-0.7)	0.6 (0.6-0.6)
**Head injury**	1.1 (1.1-1.2)	1.2 (1.2-1.3)
**Surgery**	1.6 (1.5-1.6)	2.0 (1.9-2.1)
**Hypotension**	3.0 (2.8-3.1)	1.8 (1.7-1.9)
**ISS category**		
(0–9)	**Reference**	**Reference**
(10–24)	2.5 (2.3-2.7)	3.2 (2.9-3.5)
(25–44)	4.6 (4.2-4.9)	7.3 (6.6-8.0)
(45–75)	15.5 (14.5-16.6)	26.9 (24.5-29.5)
**KAMC vs. NTDB**	2.9 (2.6-3.3)	2.3 (2.0-2.7)

**Table 3:** **Adjusted OR and 95% confidence intervals (CI) of in-hospital deaths comparing patients from King Abdulaziz Medical City (KAMC) and the National Trauma Data Bank (NTDB) by injury severity score (ISS) category, age category and by head injuries status**

ISS category ^¢^	KAMC Mortality (%)	NTDB Mortality (%)	OR (95% CI) ^^^
(1–8)	0.1	0.5	0.3 (0.0-1.2)
(9–15)	0.9	1.7	0.8 (0.5-1.2)
(16–24)	3.6	4.7	1.1 (0.7-1.6)
(25–75)	46.0	23.0	5.0 (4.3-5.8)
**Age category** ^**¥**^			**OR (95% CI)** ^**^**^
0-14	5.6	3.0	2.8 (1.6-3.1)
15-25	7.6	3.3	3.2 (2.6-3.9)
26-45	8.9	3.1	3.2 (2.5-4.0)
46-64	12.8	4.5	3.9 (2.6-5.6)
≥65	16.8	10.7	1.6 (1.0-2.6
**Head injury** ^**§**^			**OR (95% CI)** ^**^**^
No	1.9	3.8	1.9 (1.4-2.5)
Yes	17.0	6.6	3.3 (2.9-3.9)

^Interaction effects: p < 0.001.

OR Odds ratio;

^**¢**^Model covariates: Age, gender, mechanism of injury, surgery, T-RTS, hypotension, head injury, indicator variable KAMC vs. NTDB, ISS and (KAMC vs. NTDB X ISS).

^**¥**^Model covariates: Gender, mechanism of injury, surgery, T-RTS, hypotension, head injury, ISS, indicator variable KAMC vs. NTDB, age and (KAMC vs. NTDB X age).

^**§**^Model covariates: Age, gender, mechanism of injury, surgery, T-RTS, hypotension, ISS, indicator variable KAMC vs. NTDB, head injury and (KAMC vs. NTDB X head injury).

## Discussion

Our study shows that even after taking into account demographic and severity differences, injured patients hospitalized following traffic injuries in Saudi Arabia are more likely to die in the hospital than comparable patients admitted to large U.S. trauma centers. This difference was not seen in patients sustaining mild to moderate injuries, with ISSs 1–24.

We are not aware of any previous study that compared trauma mortality between Saudi Arabia and the U.S. The findings from our study are consistent with those of Mock et al., which demonstrated disparity in trauma mortality between developing and developed countries (Mock et al. [1998]). Furthermore, Perel et al. ([2012]) examined differences in trauma mortality in a large multicenter study and found patients treated in low and middle-income countries to be at higher risk of in-hospital death even after taking into account severity and demographic differences. Both of these previous studies used economic indicators as a measure of development. Although Saudi Arabia is a high-income country, it is still considered a developing country (Klugman [2011]) and resembles many low-middle income countries in terms of development indicators and infrastructure for trauma systems.

There are several potential explanations for outcome disparities found in our data for which further research is needed. It is likely that U.S. hospitals are in a better position to adopt new life-saving technologies that improve diagnosis and expedite urgent care for severely injured patients. In addition, trauma care training in the U.S. may be more advanced than programs in Saudi Arabia, which may affect the skill sets of the triage team and potentially lead to better outcomes. Although KAMC meets the criteria of a level I trauma center in the U.S., it has not gone through the formal processes of verification by either the American College of Surgeons or another entity in the United States. Therefore, it is possible that unmeasured differences in trauma resources exist in the two populations, and led to the disparity in outcome.

Higher rates of nosocomial infections (Rosenthal et al. [2012]) or limited access to blood reserves in developing countries may impact trauma mortality regardless of trauma care quality. However, it is unlikely that this is the case in Saudi Arabia because reported rates of nosocomial infection are similar to those in developed countries (Sabra and Abdel fattah [2012]) and because KAMC has a policy in place to maintain sufficient blood supply to keep up with the large patient population they serve.

Another potential explanation concerns differences in driving environments and demographics between Saudi Arabia and the U.S. There were more male patients in the Saudi registry than in the NTDB (85% vs. 63%, p < 0.001). This gender difference is a reflection of the fact that Saudi Arabia does not allow women to drive motor vehicles. In addition to skewing the sex ratio, this can potentially increase the number of occupants at the time of a traffic crash. Additionally, the average size of Saudi Arabian families is larger than U.S. families (Briana [2010]; [2012b]). Consequently, each traffic crash that occurs in Saudi Arabia is likely to injure more individuals than in the U.S. This in turn may lead to more patients requiring urgent interventions at the same time, which adds to the burden the ED has to deal with when patients are treated.

Our findings indicate the presence of effect modification by trauma severity and age. Odds ratios for mortality in KAMC were greater with higher ISS and brain injury. The absence of a mortality gradient among those with mild to moderate injuries indicates that with lower severity injuries, quality of care may not be a major factor affecting risk of mortality. Another way to see this is that, for mild and moderate injuries, KAMC performed well in terms of mortality compared to large U.S. trauma centers but disparity emerged as severity scores became higher. The possibility remains that the effect modification was due to severely injured patients at KAMC being more severe than captured by the scales. However, it seems unlikely that omitted severity components would exist that are strong enough to explain the steep gradient. This mortality difference also was unaffected by categorizing ISS score since using it as a continuous variable yielded similar results.

Unexpectedly, mean ISS values were not substantially higher for trauma patients admitted to KAMC than for those admitted to large U.S. trauma centers (12.9 vs. 12.1). The comparison may be biased if the high number of patients at KAMC led to under-triage (underestimating severity of trauma patients) (Richard Beebe [2011]). One may also speculate that the ISS difference would have been larger if the quality of medical care at the scene and during transport was comparable. If severely injured patients in Saudi Arabia were more likely to die at the scene or on the way to the hospital than in the U.S. due to a relative paucity of rapid emergency transport and skilled paramedics, average ISS among those admitted to the ER would have been lowered. On the other hand, worse pre-admission care could have increased severity in some patients. Paramedics in Saudi Arabia have Basic Life Support (BLS) training but may not be able to perform advanced life saving procedures. In addition, we found that bystanders, who mostly have no medical training, transported 37% of KAMC patients (not shown). Without further detailed study, it is difficult to assess the role of pre-admission care in the severity of admitted patients.

Because our study utilized about 10 years of hospital admissions, it is possible that clinical care has changed over the study period potentially affecting our findings. However, this would be more of a concern if the change in trauma care led to differential change in mortality in either population. When we examined in-hospital mortality in the two populations over the study period, we did not find any major trends in mortality (Figure 2).

It was not clear whether patients who arrived to the ED with CPR in progress and then pronounced dead were included as death upon arrival or as in-hospital deaths in both KAMC and NTDB. This may potentially affect our findings because counting deaths following “CPR in progress” as being in hospital would increase the estimated mortality. If this was done more frequently at KAMC, for example, we would have overestimated mortality disparity. However, it is unlikely that such considerations would have drastically affected our findings because when we excluded patients who died in the ED prior to hospital admission, the results were not very different (Table 2).

Unmeasured confounders that we were not able to address in our analysis may also have affected our findings. For example, unknown differences in the frequency and severity of preexisting conditions among the two patient populations could have contributed to the mortality differences observed. Another unmeasured confounder is population or system wide differences that could have affected patients’ trajectory after injury. Our study examined patients’ outcome after arrival to the ED. However, it is possible that population level differences between the two countries may have had a role in the patient condition prior to admission and led to differential deterioration rate between the two groups. One example of system wide difference is the distance to trauma centers, which have been found to be associated with patient outcomes (Crandall et al. [2013]; Durkin et al. [2005]).

The NTDB has potential to improve trauma care and outcomes in the U.S. (Haider et al. [2012b]; Haider et al. [2012a]) and worldwide. Although missing data, incorrect recording and data entry errors are likely to occur (Neal [2009]), its large size, detailed information and standardized structure allow answering many questions pertaining to trauma outcomes. Inviting other countries to establish similar registries has the potential to enable international collaborations and help improve trauma outcomes globally. In addition, future research utilizing NTDB for international research will contribute to existing knowledge. For example, two recent studies by Haider et al. (Haider et al. [2014]; Haider et al. [2013]) used the NTDB to shed some light on trauma outcome in other countries relative to the U.S.

Despite limitations, the results of our analysis are generalizable to patients treated in KAMC and provide some insights into the difference in trauma outcomes between Saudi Arabia and the U.S.

## Conclusion

In summary, we have demonstrated that patients injured in traffic crashes in Saudi Arabia are more likely to die after admission to the emergency department in one of the best equipped Saudi hospitals than patients admitted to large U.S. trauma centers. This excess in-hospital mortality in Saudi Arabia was present only for patients sustaining relatively severe injuries, and it was higher with increasing severity and higher age. While it is not possible to pinpoint a specific cause of the disparity, this study showed that there is room for further improvement of KAMC’s outcome. Future studies should explore reasons for outcome discrepancies among severely injured patients in order to improve the quality of care and reduce the burden of preventable mortality.

Quality improvement programs in Saudi Arabia can use these findings as a reference when examining mortality outcomes in the future to identify changes in trauma outcomes. In addition, the direct comparison approach we used in this study provides a model for future studies from other developing countries to compare outcomes with a large resource such as the NTDB.

## Authors’ original submitted files for images

Below are the links to the authors’ original submitted files for images. Authors’ original file for figure 1 Authors’ original file for figure 2

## Abbreviations

ED: Emergency Department
GCS: Glasgow Coma Scale
ISS: Injury Severity Score
KAMC: King Abdulaziz Medical City
NTDB: National Trauma Data Bank
RTS: Revised Trauma Scale
T-RTS: Triage Revised Trauma Scale
TRISS: Trauma Injury Severity Scoring
SBP: Systolic Blood Pressure
