# Supplementary figures and images for: In-hospital mortality among patients injured in motor vehicle crashes in a Saudi Arabian hospital relative to large U.S. trauma centers
Source: Inj Epidemiol. 2014 Aug 27;1(1):21. doi: 10.1186/s40621-014-0021-4 (PMC4648961; doi:10.1186/s40621-014-0021-4)

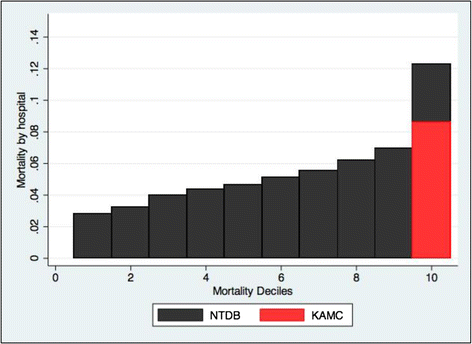

Supplement: Supplementary file 1 — Authors’ original file for figure 1 [file 40621_2014_21_MOESM1_ESM.gif]

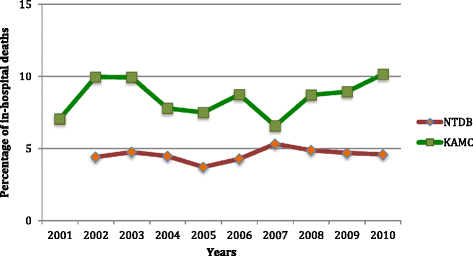

Supplement: Supplementary file 2 — Authors’ original file for figure 2 [file 40621_2014_21_MOESM2_ESM.gif]
